# Large-scale analysis of differential gene expression in coffee genotypes resistant and susceptible to leaf miner–toward the identification of candidate genes for marker assisted-selection

**DOI:** 10.1186/1471-2164-15-66

**Published:** 2014-01-24

**Authors:** Danielle C Cardoso, Juliana C Martinati, Poliana F Giachetto, Ramon O Vidal, Marcelo F Carazzolle, Lilian Padilha, Oliveiro Guerreiro-Filho, Mirian P Maluf

**Affiliations:** 1 Agronomic Institute of Campinas, Campinas, Brazil; 2 Embrapa Agriculture Informatics, Campinas, Brazil; 3 LNBio-Unicamp, Campinas, Brazil; 4 Embrapa Coffee, Brasilia, Brazil

**Keywords:** *Coffea arabica*, Leaf miner, Microarray, Plant defense

## Abstract

**Background:**

A successful development of herbivorous insects into plant tissues depends on coordination of metabolic processes. Plants have evolved complex mechanisms to recognize such attacks, and to trigger a defense response. To understand the transcriptional basis of this response, we compare gene expression profiles of two coffee genotypes, susceptible and resistant to leaf miner (*Leucoptera coffella*). A total of 22000 EST sequences from the Coffee Genome Database were selected for a microarray analysis. Fluorescence probes were synthesized using mRNA from the infested and non-infested coffee plants. Array hybridization, scanning and data normalization were performed using Nimble Scan® e ArrayStar® platforms. Genes with foldchange values +/-2 were considered differentially expressed. A validation of 18 differentially expressed genes was performed in infected plants using qRT-PCR approach.

**Results:**

The microarray analysis indicated that resistant plants differ in gene expression profile. We identified relevant transcriptional changes in defense strategies before insect attack. Expression changes (>2.00-fold) were found in resistant plants for 2137 genes (1266 up-regulated and 873 down-regulated). Up-regulated genes include those responsible for defense mechanisms, hypersensitive response and genes involved with cellular function and maintenance. Also, our analyses indicated that differential expression profiles between resistant and susceptible genotypes are observed in the absence of leaf-miner, indicating that defense is already build up in resistant plants, as a priming mechanism. Validation of selected genes pointed to four selected genes as suitable candidates for markers in assisted-selection of novel cultivars.

**Conclusions:**

Our results show evidences that coffee defense responses against leaf-miner attack are balanced with other cellular functions. Also analyses suggest a major metabolic reconfiguration that highlights the complexity of this response.

## Background

Once a plant recognizes a pathogen attack, the metabolism must balance demands for resources to support defense versus requirements for cellular maintenance, growth and reproduction [1-4]. Defense mechanisms involve a shift on metabolism, activating specific pathways such as synthesis of secondary metabolites, programmed cell death, ions translocation. Concomitantly, can occur a shutdown of other metabolic pathways not directly involved with defense response, such as those associated with growth and reproduction. A resistance and/or tolerance trait is attributed whenever this defense response is successful in controlling pathogen or herbivore attack.

Genetic control of metabolic re-programming is normally triggered by few resistance genes which are seek out to be transferred to other plants. However, resistance response involves changes in other genes, not usually identified, with determinant roles in the overall response. Therefore for an effective transference of resistance traits, to know how these genes interact during re-programming of plant metabolism is essential.

Among available methods for high-throughput analysis the microarray is a powerful tool for large-scale gene expression studies in many plant species with whole genome sequenced: potato [5,6], tomato [7,8], soybean [9,10], wheat [11], barley [12,13] maize [14,15], grape [16], pine [17], Arabidopsis [18-20]. The main advantage of microarray analyses is to evaluate the expression of large number of genes in different genotypes, organs, tissues, treatments, using the same set of genes. These genes can be compared during different biological situations allowing both an association with metabolic pathways and establishement of their role on resistance response. Several studies have been carried out using microarray analysis to identify genes associated with plant defense [21-25].

The leaf-miner, *Leucoptera coffeella* (Guérin-Méneville, 1842) (Lepidoptera-Lyonetiidae) is a specialist parasite of *Coffea* species. Upon oviposition on leaves, ecloded larvae feed directly from parenchyma tissues, leading to a reduction of foliar surface and eventual leaf drop [26]. This damage results in reduction of photosynthetic area and plant survival. In Brazilian breeding programs resistance genes from *C. racemosa* have been transferred to the susceptible *C. arabica* by controlled crosses, and so far a large number of hybrid progenies are under selection for resistance to leaf-miner [27]. Although defense mechanism to leaf-miner is not understood yet, genetic analysis demonstrated that resistance to the insect is dominant and controlled by two complimentary genes [28]. At the molecular level, there is little information regarding gene expression on coffee plants during defense response. Using subtractive hybridization methodology (SSH), Mondego et al. [29] found differentially expressed genes in coffee plants upon leaf-miner infestation, among which a *miraculin-like* encoding gene was significantly overexpressed in resistant coffee plants. Differential expression of defense-related genes such as *lipoxygenase*, *glutathione transferase*, *protein-kinase receptor* and *glucanase* was observed in response to leaf-miner infestation [30]. However, the expression profiles indicate that differences results from gene expression timing along insect infection rather than with gene regulation.

Despite the efforts of breeding programs to develop novel coffee cultivars bearing leaf-miner resistance, selection of progenies homozygotes for this trait is difficult [27], as advanced generations are still producing susceptible plants. Therefore, information regarding molecular control of resistance response as well as identification of candidate-genes associated with these processes will contribute with assisted-selection.

In this context, the aim of our study was to explore transcriptomic differences throughout insect infestation, in susceptible and resistant *C. arabica* plants challenged by *L. coffeella,* using microarray technology. The arrays were developed using coffee-specific oligoprobes designed based on gene sequences available at the Brazilian Coffee Genome Project [31]. The database contains a collection of around 32,000 gene sequences, covering most of the *C. arabica* genome [32]. Besides this, we selected a group of candidate-genes to be used as molecular markers for assisted-selection. As far as we know, this is the first report of a large-scale transcriptional profile analysis used to study gene expression changes in coffee plants in the presence of an herbivore insect.

## Results

Microarray analyses were performed to characterize large-scale gene expression profiles during leaf-miner development on coffee leaves. The analyses included a hybridization of a 135 K array with 6 different samples, corresponding to time-course infestation stages in both resistant and susceptible plants. The arrays contain sequences of around 33 K genes identified in EST libraries prepared from different physiological and metabolic situations [31]. A minimum of 6 - 24mer match probes for each selected gene were used for the array set up. The arrays were hybridized with probes corresponding to 3 treatments of both susceptible (S) and resistant (R) leaves: non-infestated (T0), after oviposition and egg-eclosion (T1) and damaged by insect feeding (T2).

Initially, differential expression patterns were identified using statistical analysis, and specific transcriptional profiles were established for each evaluated interaction. In a second approach, genes exhibiting differential expression among genotypes and treatments were submitted to *in silico* evaluations to classify and categorize those genes regarding their possible molecular functions and metabolic pathways. Finally, a group of 19 genes involved with defense-related mechanisms, exhibiting regulated expression, were further characterized using qRT-PCR.

### Microarray and statistical analysis

A total of 2141, 2359 and 2257 differentially expressed genes were identified from *in silico* analyses of raw hybridization data considering 3 interactions: T0R X T0S, T1R X T1S and T2R X T2S (Table 1). Comparing T0R X T0S we observed higher differential expression levels where foldchange values range from 400 and 1000 times in up-regulated genes, and from 200 to 400 times in down-regulated genes in resistant leaves. The other interactions exhibited foldchange values ranging from 150 and 350 times in down-regulated, and from 10 to 15 times in up-regulated genes (Table 2).

**Table 1:** Distribution of regulated genes in each interaction analyzed

**Genes**	**T0R_T0S**	**T1R_T1S**	**T2R_T2S**
	**(non infested plants)**	**(Egg hatching)**	**(Egg eclosion)**
Up regulated	1268	1231	889
Down regulated	873	1128	1368
No differences	19057	18837	18939

**Table 2:** Survey of differentially expressed genes in all considered interactions including number and fold-change values range

**T0R_T0S**	**T1R_T1S**	**T2R_T2S**	**Range of “fold change”**
14	9	10	1000-100
32	17	23	99-40
57	51	39	39-20
103	96	125	19-10
1062	1058	1171	9-2
858	1117	880	(-)2- (-)9
15	11	9	(-)10- (-)499
Total 2141	Total 2359	Total 2257	

Different regulation profiles for defense response were observed among differentially expressed genes. The first group includes *chitinase* and *polygalacturonase* genes, regulated differently in resistant and susceptible leaves from T0 to T2. The second group includes genes that were up or down regulated in control resistant or susceptible leaves, but throughout insect development inverted their expression pattern. Example of this group is the gene encoding the enzyme *polyphenol oxidase*, up regulated at T0 in resistant plants and down regulated at T1 and T2. And a third group includes genes that were up and/or down regulated in response to the leaf-miner infestation, either in susceptible or resistant leaves.

### Interaction between resistant and susceptible genotypes without insect infestation (T0R x T0S)

Since higher values for differential gene expression were observed when comparing T0R X T0S samples, we chose this interaction for further analyses and selection of candidate-genes for validation. In this interaction, 2141 genes exhibited differential expression, 1268 were up regulated and 873 were down regulated.

**Figure 1:**
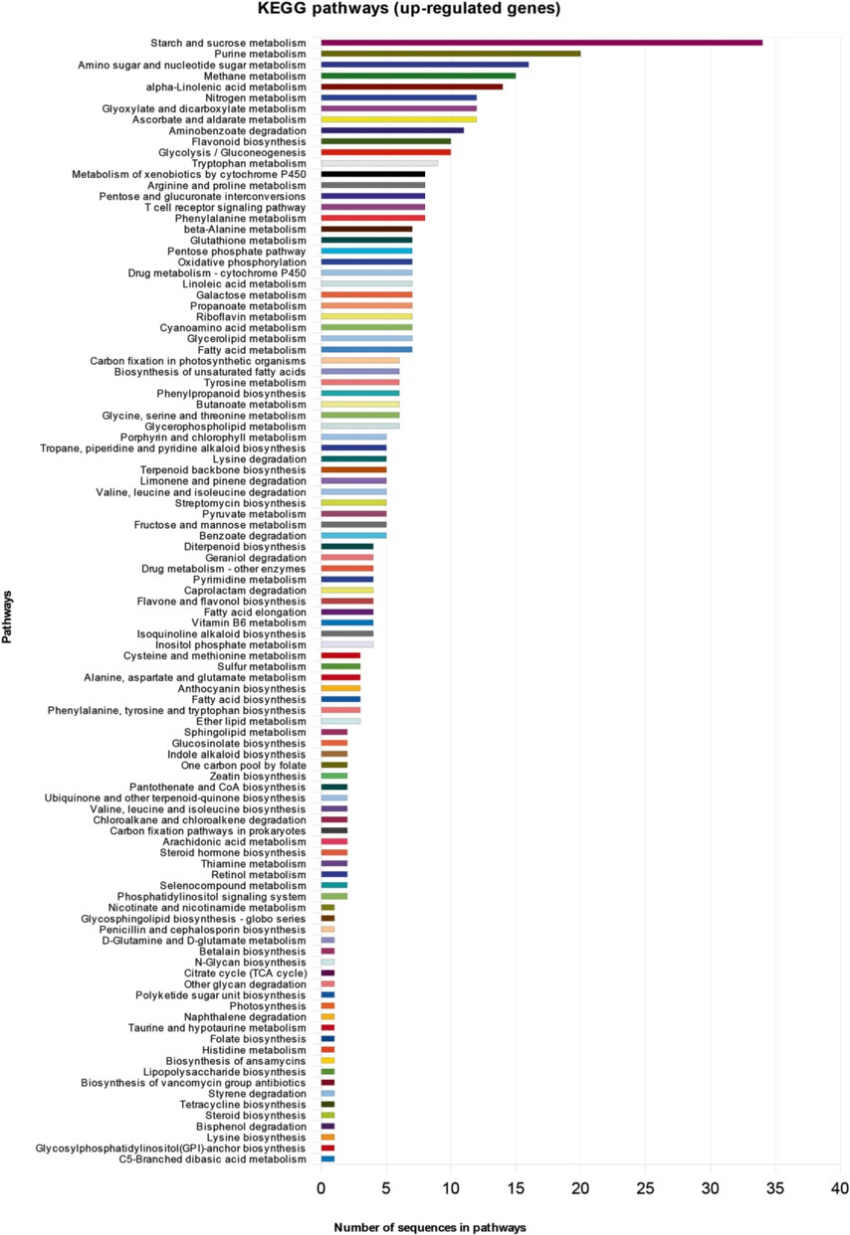
**Pathways from the top 100 up-regulated genes in T0 interaction.** Pathways were identified considering T0 interaction. Amount of genes belonging to pathways is specified in each line.

**Figure 2:**
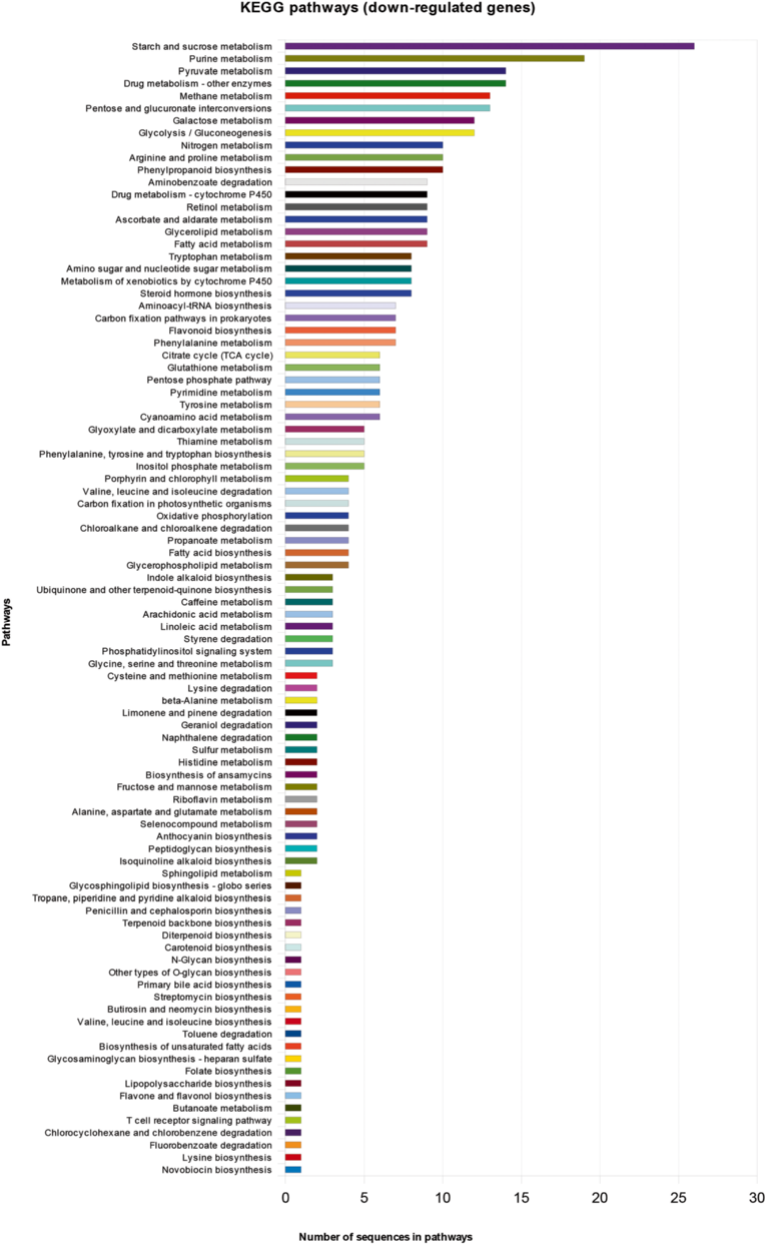
**Pathways from the top 100 down-regulated genes in T0 interaction.** Pathways were identified considering T0 interaction. Amount of genes belonging to pathways is specified in each line.

**Figure 3:**
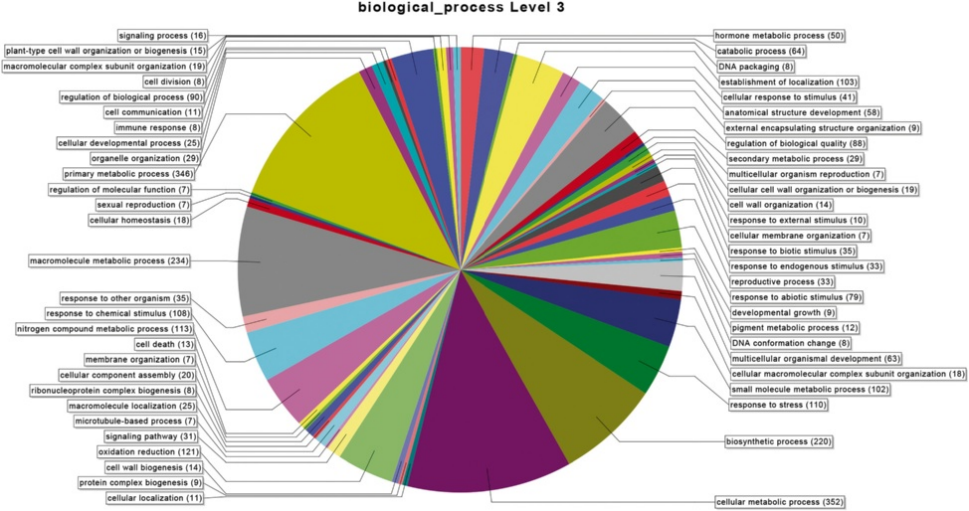
**Biological process for up-regulated genes.** Functional categorization of up regulated genes (%) in coffee genotypes (susceptible and resistant to leaf miner) without insect.

**Figure 4:**
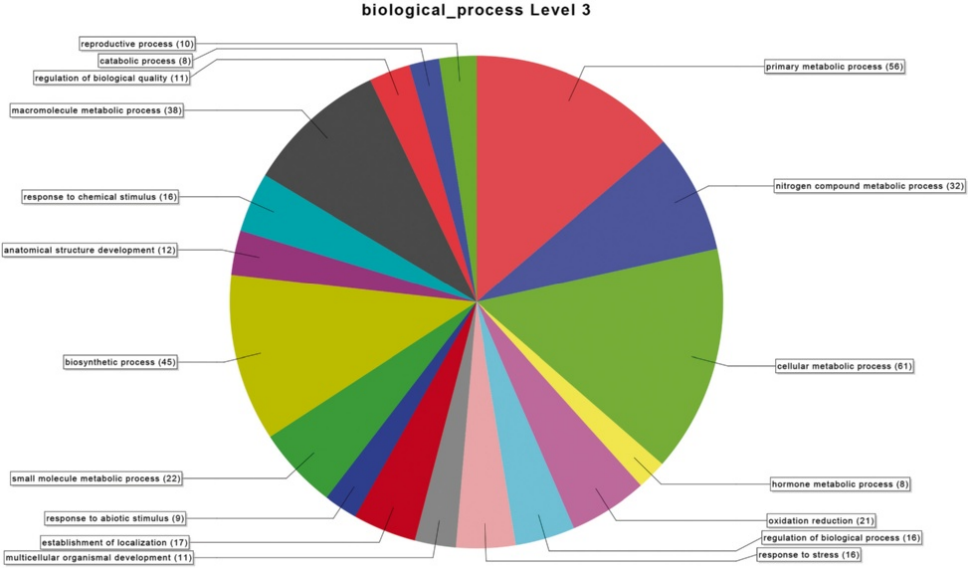
**Biological process for down regulated genes.** Functional categorization of down-regulated genes (%) in coffee genotypes (susceptible and resistant to leaf miner) without insect.

Regulated genes from the T0 samples were functionally characterized into three gene ontology categories – molecular function, component cellular and biological function - and grouped according their metabolic categories (Figures 1 and 2). A description of the first one hundred most variable genes, both up and down regulated, is shown on Tables 3 and 4 and illustrated in Figures 3 and 4. Several contigs had no correspondence with defined categories. A larger number of genes are up-regulated rather than down-regulated, and associated with primary and cellular metabolism, which included functions such as ion, protein and nucleic acid binding, hydrolase and transferase activities, among others. As these differences were observed in non-infested leaves, possibly a different transcriptional programming takes place in resistant leaves. This may result in a pre-defense status, enabling resistant plants to a faster defense response upon leaf-miner attack.

**Table 3:** List of up-regulated genes observed for the T0R_T0S interaction with respective annotation and fold-change values

**Name**	**Blast Sol network genomics**	**e-value**	Annotation*	**Fold change (T0R X T0S)**
0U1	SGN-E628893	0	---NA---	1000,27
0U2	SGN-E1352064	5e^-89^	Caffeine synthase	642,35
0U3	SGN-E1326397	0	Acidic endochitinase se2	447,45
0U4	SGN-E1310344	0	Metallothionein-like protein	280,91
0U5	SGN-E1334735	0	Kunitz trypsin inhibitor	238,17
0U6	SGN-E1316291	0	Acid phosphatase	236,53
0U7	SGN-E661231	1e^-175^	Polygalacturonase-1 non-catalytic subunit beta	236,37
0U8	SGN-E1327615	0	Kunitz trypsin inhibitor	168,12
0U9	SGN-E1337775	0	Organ-specific protein	155,92
0U10	SGN-E659257	0	Polygalacturonase-1 non-catalytic subunit beta	152,17
0U11	SGN-E1352070	0	Caffeine synthase	148,75
0U12	SGN-E642649	0	Cytokinin oxidase	143,25
0U13	SGN-E833713	1e^-146^	Asr1 protein	119,38
0U14	SGN-E667484	0	Protein	115,77
0U15	SGN-E1319644	0	pr-10 type pathogenesis-related protein	98,14
0U16	NM**		Protein	97,12
0U17	NM		Protein	94,57
0U18	SGN-E1321440	0	Acid phosphatase	93,94
0U19	NM		Invertase pectin methylesterase inhibitor family protein	82,40
0U20	SGN-E1312621	0	---NA---	76,83
0U21	SGN-E1309331	0	mpbq msbq methyltransferase 2	75,15
0U22	SGN-E837532	2e^-31^	Class iii chitinase	75,05
0U23	SGN-E1316252	0	Heat shock	73,55
0U24	SGN-E1325880	0	Swib complex baf60b domain-containing protein	72,42
0U25	SGN-E832873	1e^-119^	Kunitz trypsin inhibitor	63,26
0U26	SGN-E1322100	1e^-73^	Polyphenol oxidase	60,83
0U27	SGN-E660241	0	Polyphenol oxidase	57,50
0U28	SGN-E671322	0	60s acidic ribosomal protein p0	56,95
0U29	SGN-E1128614	8e^-11^	Protein	53,42
0U30	SGN-E682004	0	Lipid transfer protein	50,27
0U31	SGN-E1334549	0	Type ii proteinase inhibitor family protein	49,57
0U32	SGN-E1337715	0	Protein	49,57
0U33	SGN-E640935	0	Class iii chitinase	48,21
0U34	SGN-E990795	7e^-16^	Microsomal glutathione s-	47,84
0U35	SGN-E668445	0	Serine-type endopeptidase inhibitor	47,81
0U36	NM		Metallothionein-like protein	47,79
0U37	NM		Polyphenol oxidase	46,30
0U38	SGN-E640935	0	Class iii chitinase	45,63
0U39	SGN-E657601	0	Protein	44,71
0U40	SGN-E835025		4-hydroxy-3-methylbut-2-enyl diphosphate reductase	44,21
0U41	SGN-E1320197	0	Peroxisomal membrane	43,89
0U42	SGN-E636199	0	---NA---	43,72
0U43	SGN-E1333755	0	Type ii proteinase inhibitor family protein	41,88
0U44	SGN-E1337775	0	Organ-specific protein	41,68
0U45	SGN-E912118	1e^-23^	Tartrate-resistant acid phosphatase type 5	41,04
0U46	SGN-E628829	8e^-94^	Cell wall protein	40,77
0U47	SGN-E449176		Phospholipid glycerol acyltransferase family protein 7	39,90
0U48	SGN-E830846	0	r3h domain containing	39,64
0U49	SGN-E628829	8e^-94^	Oxygen-evolving enhancer protein chloroplast	39,55
0U50	NM		Auxin-independent growth promoter protein	39,13
0U51	SGN-E1349312	0	Lipid transfer protein	39,09
0U52	SGN-E639273	1e^-160^	Protein kinase domain containing expressed	38,61
0U53	SGN-E1352095	0	Protein	35,83
0U54	SGN-E838896	0	Cytochrome p450	34,98
0U55	NM		nadh dehydrogenase subunit f	34,83
0U56	SGN-E838821	0	mta sah	34,62
0U57	SGN-E669832	0	Cytokinin oxidase	33,83
0U58	SGN-E643214	0	vesicle-associated membrane protein 714	33,06
0U59	SGN-E1346029	0	Protein	32,83
0U60	SGN-E1348577	0	Protein	32,50
0U61	SGN-E674849	1e^-15^	Mitochondrial chaperonin hsp60	32,40
0U62	SGN-E788243	6e^-20^	Tartrate-resistant acid phosphatase type 5	32,40
0U63	SGN-E109388	3e^-31^	Protein	31,93
0U64	SGN-E1326628	0	Formate dehydrogenase	31,83
0U65	SGN-E1333903	0	Elongation factor-1 alpha	31,16
0U66	NM		Protein	30,56
0U67	SGN-E1321812	0	Peptidylprolyl isomerase	30,27
0U68	SGN-E1310338	0	60s ribosomal protein	30,00
0U69	NM		Conserved hypothetical protein [Ricinus communis]	29,87
0U70	SGN-E681444	0	Homeobox-leucine zipper protein	29,71
0U71	SGN-E1168096	2e^-20^	Protein	29,31
0U72	SGN-E818914	2e^-36^	Gibberellin 20	29,19
0U73	SGN-E431715	7e^-11^	Cellulose synthase	28,99
0U74	SGN-E650445	0	Poly -binding protein	28,29
0U75	SGN-E631106	3e^-19^	orf i polyprotein	28,09
0U76	SGN-E1314273	0	Ankyrin repeat domain	27,84
0U77	SGN-E1315499	9e^-23^	Atapy2 atpase nucleotide diphosphatase	27,54
0U78	SGN-E1315958	0	nadh ubiquinone oxidoreductase b14 subunit	26,38
0U79	SGN-E830665	0	sec61 transport protein	25,52
0U80	SGN-E1316141	0	Flavanone 3-hydroxylase-like protein	24,98
0U81	NM		cbl-interacting serine threonine-protein	24,92
0U82	SGN-E1342733	0	Transcription factor lim	24,70
0U83	SGN-E837532	8e^-30^	Class iii chitinase	23,94
0U84	SGN-E1322208	5e^-90^	Cysteine proteinase	23,93
0U85	SGN-E1322866	1e^-126^	Protein	23,85
0U86	SGN-E1315443	0	Acid phosphatase	23,69
0U87	SGN-E662706	0	gdsl-motif lipase hydrolase family protein	23,27
0U88	SGN-E266690	7e^-11^	Protein	23,16
0U89	SGN-E1319812	0	Protein	22,87
0U90	SGN-E1334410	0	Protein	22,82
0U91	SGN-E1321222	0	Triosephosphate isomerase	22,62
0U92	SGN-E648331	1e^-68^	Serine-threonine protein plant-	22,61
0U93	SGN-E1351186	0	Class iii chitinase	22,36
0U94	SGN-E951741	2e^-22^	Late embryogenesis abundant protein lea14-	22,06
0U95	SGN-E686943	0	Dehydrin	21,99
0U96	SGN-E674268	1e^-90^	mta sah	21,79
0U97	SGN-E1352075	0	7-methylxanthine n-methyltransferase	21,73
0U98	SGN-E1323598	0	Transcription initiation factor iib	21,29
0U99	SGN-E1348381	1e^-151^	Translation factor	21,07
0U100	SGN-E1322408	0	Beta-glucosidase-like protein	20,89

*The annotation of each sequence was established on the Coffee Genome Database [31].

**No match with any sequence on the Solanaceae Genomic Database.

**Table 4:** List of down-regulated genes observed for the T0R_T0S interaction with respective annotation and fold-change values

**Name**	**Blast Sol network genomics**	**e-value**	Annotation*	**Fold change (T0R X T0S)**
0D1	SGN-E1320843	0	Protein	-445,87
0D2	SGN-E676870	0	Protein	-235,45
0D3	SGN-E1320843	0	Zinc finger	-126,65
0D4	SGN-E1325444	0	Hypothetical protein VITISV_000181 [Vitis vinifera]	-62,54
0D5	SGN-E835732	0	Tapetum-specific protein lla-115	-49,63
0D6	SGN-E661762	0	---NA---	-35,43
0D7	NM**		PREDICTED: hypothetical protein [Vitis vinifera]	-24,73
0D8	NM		Glycerol-3-phosphate acyltransferase 6	-17,93
0D9	SGN-E1321887	0	gdsl-motif lipase hydrolase-like	-16,98
0D10	SGN-E1033676	5e^-19^	er glycerol-phosphate acyltransferase	-16,50
0D11	SGN-E1322050	0	Extensin-like protein	-14,88
0D12	SGN-E791894	1e^-118^	Cytochrome p450	-12,96
0D13	SGN-E837009	0	Cytochrome p450	-11,39
0D14	NM		Cytochrome b	-11,12
0D15	SGN-E1349845	1e^-137^	gdsl-motif lipase hydrolase family protein	-10,27
0D16	SGN-E1318049	1e^-153^	Gibberellin-regulated protein 1	-9,89
0D17	SGN-E660879	0	Zinc finger	-9,88
0D18	SGN-E835266	0	Isocitrate lyase	-9,22
0D19	SGN-E673783	0	Isocitrate lyase	-8,84
0D20	NM		---NA---	-8,83
0D21	SGN-E660879	0	Zinc finger	-8,73
0D22	SGN-E680272	1e^-128^	Protein	-8,498
0D23	SGN-E898278	4e^-13^	Protein	-8,43
0D24	SGN-E1328871	0	---NA---	-8,16
0D25	NM		Cytochrome p450	-8,02
0D26	SGN-E678498	0	Protein	-7,36
0D27	SGN-E1319644	0	Serine-threonine protein plant-	-7,35
0D28	SGN-E838812	1e^-40^	---NA---	-7,34
0D29	NM		Pathogenesis-related protein 1	-7,27
0D30	SGN-E1312882	0	Heat shock protein	-7,17
0D31	NM		---NA---	-7,12
0D32	SGN-E830806	0	Cytochrome p450	-7,06
0D33	SGN-E1334002	0	abc transporter	-7,00
0D34	SGN-E659349	0	Glutathione s-transferase gstu6	-6,85
0D35	SGN-E1317104	0	Aspartyl protease family protein	-6,74
0D36	SGN-E1322588	1e^-180^	at1g72120 f28p5_2	-6,53
0D37	NM		---NA---	-6,46
0D38	SGN-E1350292	0	Lactoylglutathione lyase family protein	-6,46
0D39	NM		Achain crystal structure of a cell-wall invertase from Arabidopsis thaliana in complex with sucrose	-6,36
0D40	SGN-E1335955	3e^-56^	Retroelement pol polyprotein	-6,24
0D41	SGN-E820310	2e^-64^	Xyloglucan endotransglucosylase hydrolase protein 22	-6,23
0D42	SGN-E836814	1e^-169^	Leucine-rich plant specific	-6,20
0D43	SGN-E686810	0	Zinc finger	-6,09
0D44	SGN-E839045	0	Glucose-methanol-choline oxidoreductase family protein	-6,06
0D45	SGN-E531670	1e^-12^	Hydrolyzing o-glycosyl	-5,97
0D46	SGN-E1216540	2e^-27^	Aminotransferase family protein	-5,91
0D47	SGN-E747084	4e^-68^	Alkaline alpha-galactosidase seed imbibition protein	-5,81
0D48	SGN-E836814	1e^-179^	Leucine-rich plant specific	-5,71
0D49	SGN-E1345225	0	---NA---	-5,71
0D50	SGN-E1325272	0	Protein	-5,69
0D51	SGN-E626178	8e^-17^		-5,62
0D52	NM		Alkaline alpha-galactosidase seed imbibition protein	-5,60
0D53	NM		Cinnamoyl reductase-like protein	-5,57
0D54	SGN-E1349228	0	Proline dehydrogenase	-5,54
0D55	SGN-E775239	8e^-14^	Kinesin like protein	-5,46
0D56	NM		Methyl-accepting chemotaxis sensory transducer	-5,45
0D57	NM		Transcription factor	-5,42
0D58	NM		3-hydroxyisobutyrate dehydrogenase family protein	-5,39
0D59	NM		Outer membrane porin protein	-5,36
0D60	SGN-E1317853	0	Transcription activator	-5,31
0D61	SGN-E666413	0	bahd family clade v	-5,20
0D62	SGN-E658983	0	Glycerol-3-phosphate dehydrogenase	-5,18
0D63	SGN-E1327315	0	Tonoplast intrinsic	-5,17
0D64	SGN-1331462	0	Achain crystal structure of a cell-wall invertase from arabidopsis thaliana in complex with sucrose	-5,08
0D65	NM		Inner-membrane translocator	-5,00
0D66	NM		Beta-ig-h3 fasciclin	-4,94
0D67	SGN-E653486	1e^-52^	bahd family clade v	-4,90
0D68	NM		Stachyose synthase	-4,86
0D69	SGN-E1349101	3e^-46^	60s acidic ribosomal protein p1	-4,85
0D70	NM		Disease resistance	-4,79
0D71	SGN-E666413	0	bahd family clade v	-4,75
0D72	SGN-E1313854	0	Cytosolic aldehyde dehydrogenase	-4,75
0D73	SGN-E1320568	0	Protein	-4,72
0D74	SGN-E748200	1e^-22^	Anthranilate synthase alpha subunit	-4,71
0D75	SGN-E1316428	0	Heat shock protein	-4,68
0D76	NM		Protein	-4,67
0D77	SGN-E700960	8e^-56^	Magnesium transporter	-4,67
0D78	SGN-E1321133	0	Glutathione s-transferase	-4,66
0D79	SGN-E1309644	1e^-33^	---NA---	-4,66
0D80	NM		nac domain ipr003441	-4,63
0D81	SGN-E791702	0	Zinc finger	-4,57
0D82	SGN-E524668	0	Protein	-4,49
0D83	SGN-E667829	0	gdsl-motif lipase hydrolase family protein	-4,46
0D84	SGN-E955597	4e^-15^	Proline dehydrogenase	-4,44
0D85	NM		---NA---	-4,43
0D86	SGN-E1312314	0	PREDICTED: hypothetical protein [Vitis vinifera]	-4,39
0D87	NM		Undecaprenyl pyrophosphate phosphatase	-4,37
0D88	SGN-E1350610	0	Cytosolic class i small heat-shock protein	-4,35
0D89	NM		---NA---	-4,34
0D90	SGN-E747084	2e^-89^	Alkaline alpha-galactosidase seed imbibition protein	-4,34
0D91	NM		Protein	-4,32
0D92	SGN-E528554		pili assembly chaperone	-4,31
0D93	SGN-E528554	7e^-30^	Cell-wall invertase	-4,28
0D94	NM		nbs-lrr resistance protein	-4,26
0D95	NM		Transcriptional family	-4,26
0D96	NM		---NA---	-4,24
0D97	NM		Oxysterol binding protein	-4,21
0D98	SGN-E1196563	2e^-53^	ap2 domain-containing transcription factor	-4,20
0D99	SGN-E834183	0	nac domain protein nac2	-4,19
0D100	SGN-E678677	0	---NA---	-4,19

*The annotation of each sequence was established on the Coffee Genome Database (Vieira et al., 2006).

**No match with any sequence on the Solanacea Genomic Database.

### Metabolic pathways

The categorization of annotated genes using Kegg database was performed with the first 100 up and down regulated genes (Figures 1 and 2). Most of these genes are from starch and purine metabolism, and several others are involved in primary metabolism. Three main metabolic pathways are highlighted here: citrate metabolism, linoleic acid metabolism and phenylpropanoids metabolism.

**Figure 5:**
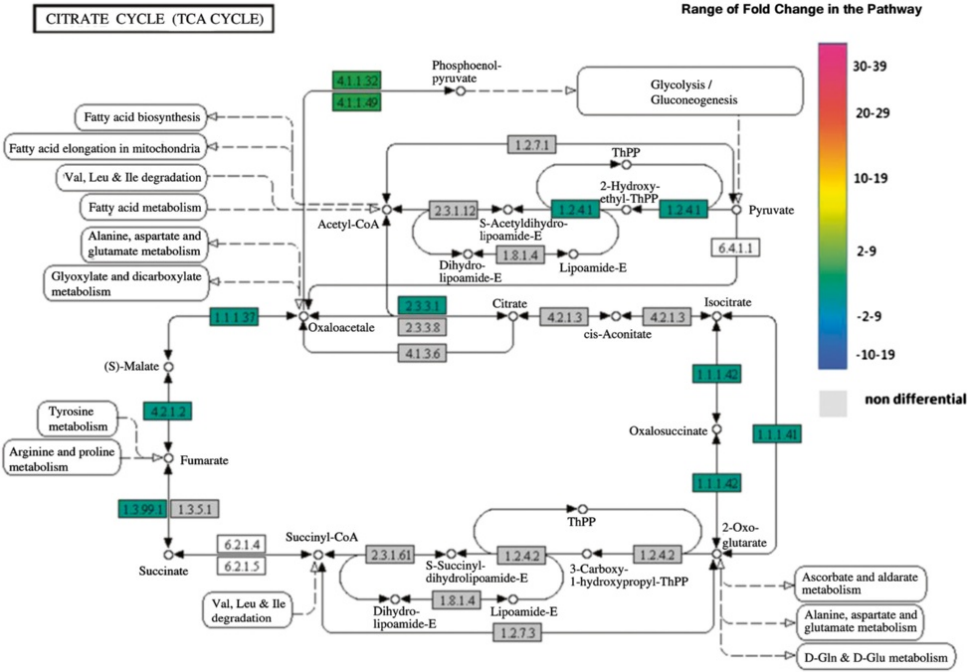
**Citric acid pathway (KEGG database).** Enzymes are identified by E.C. number and those corresponding to differentially expressed genes are highlighted according to the color scale. Color codes for each gene at T0 are as follows: red for up-regulated, green for down-regulated and grey for no differential expression. **E.C number and correspondent enzyme**: **1.1.1.37** - Malate dehydrogenase; **1.1.1.41** - Isocitrate dehydrogenase (NAD(+); **1.1.1.42** -Isocitrate dehydrogenase (NADP(+); **1.2.4.1** - Pyruvate dehydrogenase (acetyl-transferring) (Pyruvate dehydrogenase) **1.3.99.1** - Succinate dehydrogenase (Fumarate reductase); **2.3.3.1** - Citrate (Si)-synthase, **4.1.1.32** – Phosphopyruvate carboxylase; **4.2.1.2** – Fumarate hydratase (Fumarase).

We choose the citric acid cycle for further characterization as previous analyses using NMR indicated that lower levels of malate, a metabolite resulting from the citrate metabolism, are observed in resistant coffee leaves [33]. Several genes encoding citric acid cycle enzymes exhibit differential-expression (Figure 5). Expression of *isocitrate lyase* gene is repressed in resistant genotypes at T0 (fold change value -8,84), suggesting that synthesis of malate may be deficient, and therefore low levels of malate may accumulate in those leaves. However, this gene is up-regulated upon oviposition and egg ecclosion (fold change value 2,33).

**Figure 6:**
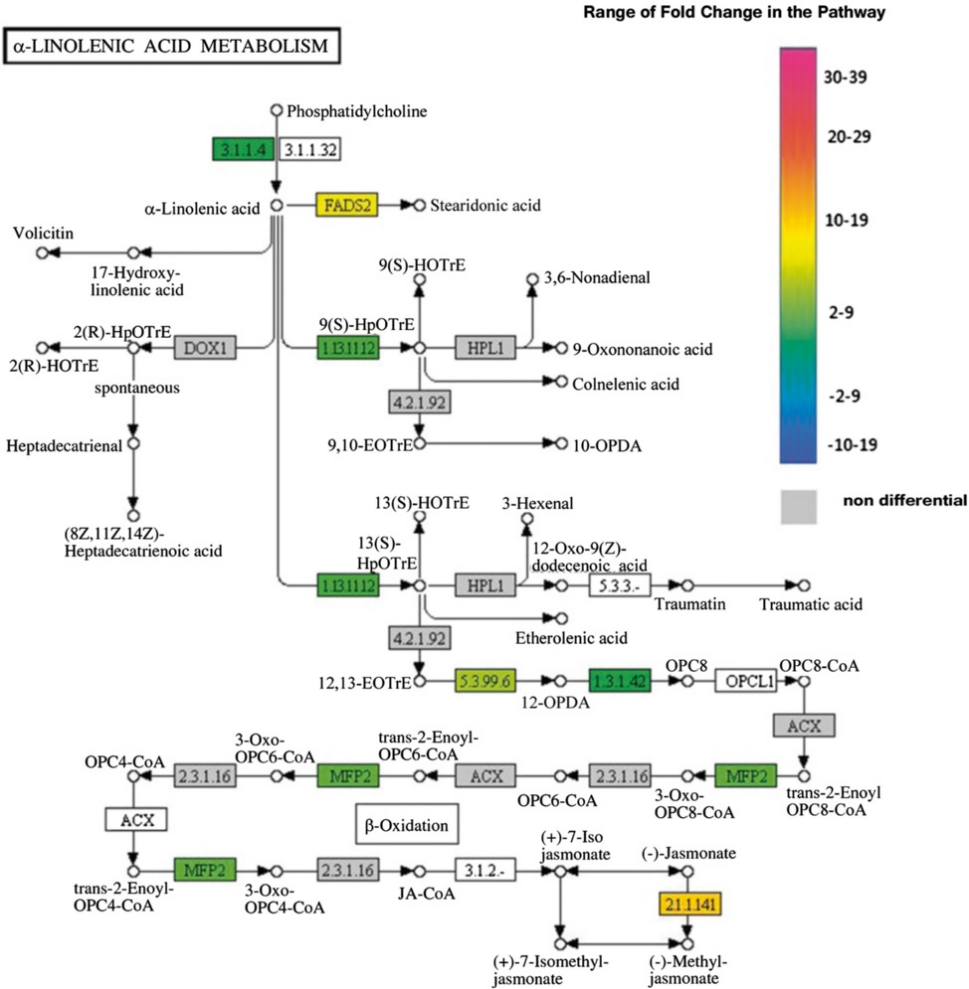
**Alfa-Linolenic acid metabolism pathway (KEGG database).** Enzymes are identified by E.C. number and those corresponding to differentially expressed genes are highlighted according to the color scale. Color codes for each gene at T0 are as follows: red for up-regulated, green for down-regulated and grey for no differential expression. **E.C number and correspondent enzyme: 1.3.1.42** – 12-oxophytodienoate reductase; **3.1.1.4 –** Phospholipase A2; **1.13.11.12** – Linoleate 13S-lipoxygenase; **5.3.99.6** – Allene oxide cyclase; **2.1.1.141** - Jasmonate O-methyltransferase**; FADS2** – fatty acid desaturase 2 ; **MFP2** – 3-hydroxyacyl-CoA dehydrogenase/ enoyl-CoA hydratase.

The linoleic acid is the first substrate of the Jasmonic acid (JA) pathway, a major signaling pathway during herbivore-defense responses. Control resistant plants (T0) show up-regulation of *jasmonate O-methyltransferase* and *lipoxygenase* while differential expression for these genes was not observed at any stage in susceptible genotypes (Figure 6). Also, 13 genes from the alpha-linoleic acid metabolism and 57 genes from jasmonate biosynthesis were regulated in resistant plants (Figure 6). For instance, transcripts of *enoyl-CoA hydratase* and *phospholipase A2* were four times more expressed in T0 than in T1 in resistant genotypes (Figure 6), but increased only at later stages in susceptible leaves.

**Figure 7:**
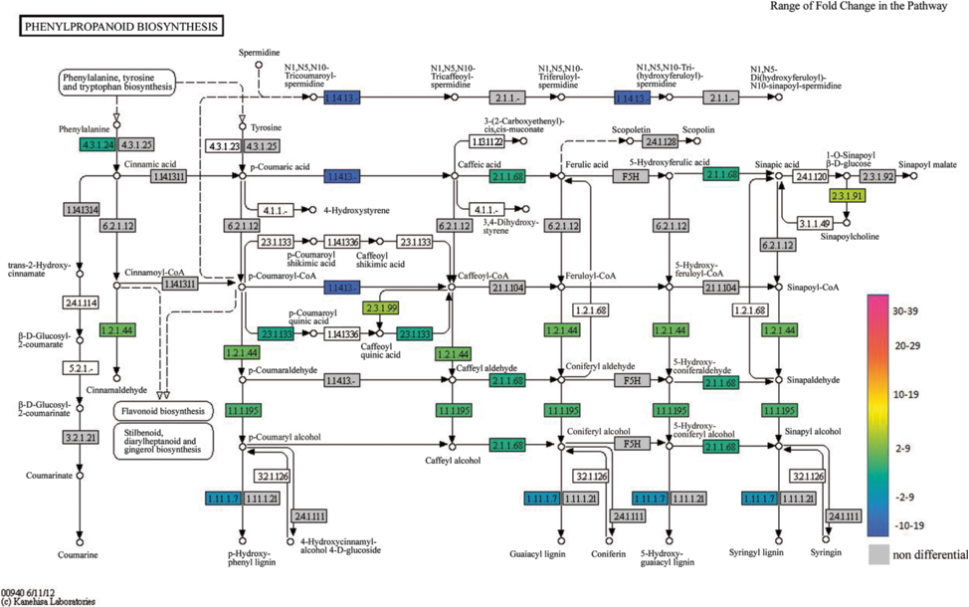
**Phenylpropanoid biosysthesis pathway (KEGG database).** Enzymes are identified by E.C. number and those corresponding to differentially expressed genes are highlighted according to the color scale. Color codes for each gene at T0 are as follows: red for up-regulated, green for down-regulated and grey for no differential expression. **E.C number and correspondent enzyme: 1.1.1.195** - Cinnamyl-alcohol dehydrogenase; **1.2.1.44** - Cinnamoyl-CoA reductase; **1.11.1.7** - Peroxidase; **1.14.13** monoxygenase; **2.1.1.68** Caffeate O-methyltransferase; **2.3.1.91 -** Sinapoylglucose--choline O-sinapoyltransferase **2.3.1.99 -** Quinate hydroxycinnamoyltransferase, **2.3.1.133 -** Shikimate O-hydroxycinnamoyltransferase; **4.3.1.24** - Phenylalanine ammonia lyase.

Phenylpropanoids are major plant phtytoalexins, part of the secondary metabolism (Figure 7). Twenty-seven genes from phenylpropanoids synthesis exhibited differential expression at T0, with foldchange values ranging from 9 to -5. Transcript levels of *phenylalanine ammonia lyase* (PAL), the enzyme that catalyzes the first step of the pathway, is up-regulated only at T0 in resistant plants (2,05), and this level decreases along insect development. In susceptible plants, PAL transcript levels increase at final steps of insect infection, T2 (2,55). However, genes fromlignin and isoflavones synthesis, downstream metabolites, such as *cynnamyl alcoholdehydrogenase* (-2,79) and *isoflavone reductase* (-1,20)*,* are down-regulated in resistant plants. On the other hand, genes from biosynthesis of anthocyanins and tannins, such as *flavonoide 3’-hydroxylase* (T0 = 24; T1 = 5; T2 = 8) and *leucoanthocyanidin dioxygenase* (T0 = 2; T1 = 9; T2 = 3) are up-regulated at all times in resistant plants. This expression profile suggests that synthesis of anthocyanins and tannins is favored over synthesis of flavones. Also, activation of upstream genes such as *phenylalanine ammonia lyase*, *chalcone synthase* and *flavonone dehydrogenase* is observed at final stages in susceptible plants, indicating that phenylpropanoid biosynthesis is delayed.

Several other defense-related genes are also positively regulated in resistant plants, including herbivore-response related genes *glutathione-S-transferase* and *cysteine proteinase inhibitor*. Apoptosis-related genes have a variable expression profile: *catalase* is up-regulated throughout insect development but *citocrome c oxidase*, *superoxide dismutase* and a *senescence-associated protein* have no differential expression, and *polygalacturonase* is up-regulated only at T0.

### Validation of expression profile for selected candidate-genes

We selected 18 genes for validation, listed with corresponding expression levels on Table 5. Genes exhibited a consistent expression pattern when quantified by either microarray or qPCR, and the Pearson coefficient for this comparison is 0.92

**Table 5:** Expression Pattern of eighteen genes selected for validation

**Name**	**Blast SOL network genomics**	**Annotation**	***Fold-change*** value^*^(microarray)	ΔΔCt value^**^(relative expression)	Coef. Pearson.***
0U17	NM	Protein	94	39	0,9242
0U6	SGN E1316291	Acid phosphatase	236	15	
0U14	SGN E667484	Protein	115	36	
0U2	SGN E1352064	Caffeine synthase	642	179	
0U4	SGN E1310344	Metallothionein-like protein	280	8,45	
0U322	SGN E450221	nadp-dependent d-sorbitol-6-phosphate dehydrogenase	6,56	3	
0U1	SGN E628893	No hits	1000	186,9	
0U42	SGN E636199	---NA---	43	8,16	
0D6	SGN E661762	---NA---	-35	-4,3	
0D10	SGN E1033676	Glycerol-phosphate acyltransferase	-16	-1,8	
0D2	SGN E676870	Protein	-235	-120	
0D1	SGN E1320843	Protein	-445	-200	
0D19	SGN E673783	Isocitrate lyase	-8	-6	
0D3	SGN E1320843	Zinc finger	-126	-17	
0D4	SGN E1325444	Hypothetical protein	-62	-36	
0D8	NM	Glycerol-3-phosphate acyltransferase 6	-17	-2,3	
0D9	SGN E1321887	gdsl-motif lipase hydrolase-like	-16	-2,2	
0D7	NM	Hypothetical protein	-24	-5,8	

^*^Microarray analysis value.

**qRT-PCR analysis value.

***Pearson coefficient correlation value.

**Figure 8:**
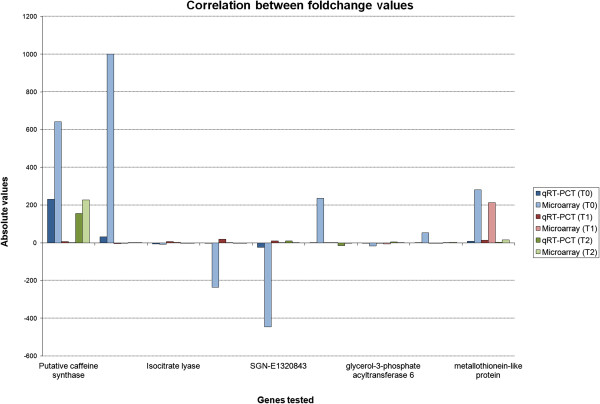
**Correlation between foldchange values obtained from microarray analysis and qRT-PCR.** Blue bars indicate values for T0 interaction (T0R_T0S), red bars for T1 interaction (T1R_T1S), green bars for T2 interaction (T2R_T2S). Dark colors of each interaction indicate fold change values from microarray analysis and soft color from qRT-PCR.

Further qPCR analyses were performed to validate expression of selected leaf-miner resistance-associated candidate-genes (Figure 8). These included genes from pathways described above and genes with either no significant hits or similarity to unknown proteins, which may represent coffee specific genes, not yet identified or characterized.

The *putative caffeine synthase* gene exhibited the greatest response to leaf-miner infection (Figure 8), as its expression was significantly higher (Relative Quantification value – RQv 230.45) in control resistant leaves, and also in later stages (RQv 155.24). The expression of gene SGN-E628893, encoding an unknown protein, is significantly higher (RQv 1000) in control resistant leaves than in susceptible ones (Figure 8). However, transcript levels dropped in resistant plants at first stages of infection, such as oviposition (RQv -1.53) and egg ecclosion (RQv 1.22). This gene is a good candidate for differentiation of resistant and susceptible plants, although possibly is not related to resistance.

The *isocitrate lyase* gene is down-regulated (RQv -6.38) in resistant leaves at T0. At initial steps of infection its transcript levels increased rapidly (RQv 5.49), but decreased during feeding stages. Other evaluated genes also exhibited a regulated expression. The *glycerol-3-phosphate acyltransferase 6* gene, a trans-membrane protein associated with synthesis of cutin, is up regulated (RQv 11.12). A gene encoding *metallothionein-like protein*, a class of metal-chelators proteins with possible anti-oxidant role, is also up-regulated (RQv 12.88) at initial stages of insect development.

Genes encoding unknown proteins with lipase protein domain, such as SGN-U585128, SL2.40ch08 and SGN-U585128 were activated in resistant plants, being up-regulated at oviposition and/or egg ecclosion with RQ values of 19.72, 19.03 and 10.30 respectively. As these genes are down-regulated on leaves at T0, this profile during insect development suggests a role during defense response (Figure 8).

The expression profile of *polygalacturonase* gene was not confirmed by real-time PCR. According to microarray *in silico* analysis this gene is activated at T0 (fold change value 236,370), and afterwards transcript levels drop along insect development (fold change value 1,628). Yet by real-time PCR analysis transcript levels are similar in susceptible and resistant plants at all times. This result may reflect differences on genetic background of evaluated plants, once they are part of a population still segregating for some characteristics. However, as this was the only observed discrepancy between all performed analyses, these genetic differences may not be associated with the resistance trait.

## Discussion

The use of resistant or tolerant cultivars represents an alternative for reducing the use of chemical defensives, the costs of production, and the negative impact over environment. In coffee, breeding for herbivore resistance is important once *Coffea arabica*, the main commercial *Coffea* species, is susceptible to almost all known coffee pathogens. Then resistance must be transferred from other compatible species, which is not always possible due to limited efficiency of inter-specific crosses.

At the Agronomic Institute (Campinas, Brazil) leaf-miner resistance genes have been transferred from *C. racemosa* to *C. arabica* through traditional breeding strategies, and although the program is currently at advanced generations, no resistant commercial cultivar is available yet. The lack of knowledge of molecular aspects controlling the resistance response, and the restricted genetic variability of breeding populations [34] limit the development of genomic-based selection tools. In this context, we aimed to provide information on molecular aspects of leaf-miner defense mechanisms and identification of reliable candidate-marker genes for assisted-selection. Those genomic tools associated with traditional breeding strategies guarantee that agronomical traits such as productivity and cup quality will be selected in advanced generations. Also, once novel genomic-markers associated with other desirable traits are developed for coffee genotypes, a genome-wide selection strategy will be possible to develop multiple-pathogen resistant cultivars. We chose the microarray analysis approach to assess the co-expression of a large amount of genes, including those that are not looked at in common analyses. Nevertheless, the results described in this work indicated that pathways regularly activated in response to herbivory, such as linoleic acid cycle, phenylpropanoids synthesis and apoptosis, are also activated during coffee-response to leaf-miner. Genes associated with jasmonate (JA) synthesis, such as *lipoxygenase* and *enoyl-CoA hydratase*, and with flavonoids synthesis, such as *chalcone synthase* and *flavanone 3-hydroxylase-like*, are up-regulated in resistant plants. Also, pathways from the primary metabolism, such as the citric acid cycle are down-regulated during leaf-miner defense response in resistant plants, a profile observed also in conifers [35].

Results of this study provide evidence that most genes encoding enzymes from the citric acid cycle are down-regulated in resistant plants (Figure 5). In a parallel analysis, a metabolite profile was established for resistant and susceptible genotypes using an NMR-based technique [33] and indicated that malate levels on resistant leaves are lower than in susceptible ones. Malate results from conversion of either fumarate or glyoxylate. Expression of *fumarase*, that converts fumarate into malate, is down-regulated at T0 in resistant genotypes (-2), what could explain the low malate levels. Production of malate from glyoxylate may also be deficient in resistant plants, once genes encoding for *malate synthase*, that converts glyoxylate into malate, and for *isocitrate liase*, the upstream enzyme that converts isocitrate into glyoxylate and succinate, are both down-regulated (-2,51 and -9, respectively). In contrast to this profile, susceptible plants exhibit a regular expression levels for these genes at T0. Therefore, both metabolic and transcriptional profiles support the affirmations that citrate cycle is down regulated in leaf-miner resistant coffee plants, and the model of down-regulation of primary metabolism in herbivore-resistant plants [36].

Biosynthesis of JA starts with alpha-linoleic acid release in non-injured tissues, triggered by *systemin* and *phospholipase A2.* Alpha-linoleic acid is then converted to JA after enzymatic steps performed by *13-lipoxygenase* (LOX), *allene oxide synthase* (AOS), *jasmonate o-methyltransferase* and others [37,38]. Several genes from the JA biosynthesis pathway are up-regulated in resistant plants at T0, including those from downstream steps such as *jasmonate o-methyltransferase* which expression is 10-fold higher than in susceptible plants. All genes of the JA biosynthesis are either down-regulated or up-regulated at later stages in susceptible plants, as for instance expression of LOX (T0 -8.66; T1 1; T2 2) increases only at T1. These observations suggest that the JA signalling pathway, including intermediate signaling-molecules such as oxo-pentenyl-cyclopentane (OPC), may be impaired in susceptible plants. Down-regulation of genes from later steps of JA biosynthesis, such as *allene oxide cyclase*, *allene oxide synthase, carboxyl methyltransferase,* the enzyme that converts jasmonic acid into methyl jasmonate, is observed at T1 and T2 in resistant plants. Therefore, a feedback regulation may be activated, with a re-programming of transcriptional response upon leaf-miner infection.

Genes associated with biosynthesis of secondary compounds are shown here to be regulated. Expression profile of genes from phenylpropanoids biosynthesis, both up-stream genes such as PAL and CHS, and downstream genes such as *flavonoide 3’-hydroxylase*, *leucoanthocyanidin dioxygenase*, reveals a preferential synthesis of tannins and anthocyanins instead of ligninin, flavones and isoflavones in resistant plants. This profile indicates a direct defense strategy against leaf-miner, once among anthocyanins and tannins are found toxic compounds with antifeedants effects over insects [39,40].

Another gene linked to secondary metabolism is a *putative caffeine synthase*, which encodes one enzyme from caffeine biosynthetic pathway. Caffeine is an alkaloid distributed in coffee plant tissues and organs. The fact that expression of a gene from its biosynthesis is significantly increased upon leaf-miner infection suggests that caffeine may have a role in defense response. However, several studies regarding caffeine and leaf-miner development indicated that this compound has no effect on insect survival rates [41,42].

Once the ultimate goal of this study is to identify potential candidate for markers, several genes were selected for validation using real-time PCR. Potential candidates include: *isocitrate lyase*, which increased expression during initial steps of leaf-miner infection may be co-related with reduction of primary carbon metabolism; *putative caffeine synthase*, part of an important pathway of coffee plants; *glycerol-3-phosphate acyltransferase 6*, a gene associated with lipid metabolism and part of cutin biosynthetic pathway, a secondary metabolite [43]; and finally *metallothionein-like protein* gene, a metal-transporter protein family with an uncertain role in plant metabolism but previously associated with redox responses [44]. Future analyses include cloning and re-sequencing genomic regions of target genes from different genotypes in order to identify suitable polymorphisms.

Among selected genes are those that have no similarity with any known reported gene or protein. Although they could not yet be associated with a biological process, their expression profile was very specific and related to defense response. For instance, genes SGN-E676870, SGN-E1128614 SGN-E1320843 were activated upon leaf-miner infection in resistant plants, and therefore represent good candidates for further investigation. Another interesting unknown gene is SGN-E628893, which is highly activated in resistant plants at T0 but is repressed upon infection. The expression profile indicates that this gene is useful for early differentiation between resistant and susceptible plants.

In summary, differential expression profiles between resistant and susceptible genotypes are observed even in the absence of leaf-miner, indicating that defense is already build up in resistant plants, as a priming mechanism. Then, a systemic defense response may be more rapidly activated in resistant plants, once basic compounds such as nitrogen and sugars are readily available as a result of repression of primary metabolism. This shift in plant metabolism is common after a pathogen attack, where defense-related pathways are activated, resulting in reduction of growth and reproduction, and in changes on link-source relationship [45]. During herbivore-defense transcript levels of genes involved in photosynthesis are also down-regulated [21,46], probably as a strategy to liberate nitrogen compounds for the secondary metabolism. Maintenance of these physiological and metabolic states has a high energetic cost, and could represent a survival limitation if nutritional conditions on the field are depleted. Actually, field observations in cultivated areas demonstrated that leaf-miner resistant coffee plants, under a severe nutritional deficit, are attacked by the leaf-miner at the same intensity as susceptible plants [27].

## Conclusions

As a basal defense state is decisive for triggering a rapid resistance response, genes associated with priming validated here, represent key genes for assisted-selection. Future studies will focus on comparisons of selected genes genomic sequences, from both resistant and susceptible parental lines, to identify suitable marker polymorphisms.

## Methods

### Plant materials

Resistant and susceptible coffee progenies were developed by the Coffee Breeding Program from the Agronomic Institute (IAC), Campinas, São Paulo, Brazil. The evaluated population (H14954-46), with 136 plants, is a F_2_BC_5_ generation of the inter-specific cross (*C. racemosa* X *C. arabica*) X *C. arabica*.

Plants were evaluated regarding the defense-response to *Leucoptera coffeella* using infestation methodology described by Guerreiro-Filho et al. [28]. After egg hatching, 1.8 cm leaf discs were taken from the leaves using a cork bore. Discs were placed on damp plastic foam and maintained in a plastic box for two weeks. Resistance/susceptibility response was visually scored according to the evaluation scale defined by Ramiro et al. [26].

### Coffee leaf miner infestation of selected plants

Fifteen resistant (R) and fifteen susceptible (S) coffee plants previously selected were used for leaf-miner infestation. Plants/seedlings of each group (R and S) were challenged with *L. coffeella* in rearing cages and following the same procedures described above. Control non-infestaded plants of each group were also evaluated. Three independent infestation experiments were used for further analyses.

Leaves were collected from the third and fourth pair from plants during different stages of insect development. Stages corresponded to egg hatching, after 1 to 5 days after infestation with *L. coffeella* (T1), and egg eclosion and tissue injury, after 6 to 10 days after infestation (T2). Control non-infested leaves (T0) were also collected for each genotype. Three leaves of each plant and each stage of insect development were collected (totalizing nine leaf per time of sampling/genotype) and immediately frozen in liquid nitrogen and stored at -80°C until RNA extraction. Experimental design was completely randomized including three replicates for each sample.

### RNA isolation and preparations

Total RNA for both NimbleGen microarray hybridization and real-time qPCR experiments was isolated using protocol described by Chang et al. [47]. RNA extractions were performed using 2 g of tissue of pooled samples. All RNA samples were analyzed by formaldehyde-agarose gel electrophoresis and by spectrophotometry to assess physical and chemical integrity. To avoid contamination by polyphenols, carbohydrates and proteins, only RNA samples with OD 260/280 and 260/230 > 1.8 were selected for further analysis. For microarray hybridizations, extracted RNA was also checked for purity and degradation using an Agilent Bioanalyzer 1000 (Agilent Technologies). Samples were stored at -80°C until further use.

### cDNA double strand synthesis, labeling and hybridization

Ten thousands nanograms (10.000 ng) of each RNA sample were pooled and treated with DNAse- RNAase free for cDNA synthesis and labelling. Three biological replicates of each treatment were used for hybridization with the cDNA microarray chip. Equal amounts of each replicate from resistant and susceptible plants were pooled respectively to minimize variation between individual RNA samples. All RNA samples were sent to Roche NimbleGen Systems, where cDNA synthesis and Cy3 labeling were performed following the manufacturer’s procedures (Nimblegen Gene Expression Analysis protocol, Nimblegen Systems, Inc., Madison, WI, USA). Equal quantities of total RNA of each sample were converted to double strand cDNA (cDNA Synthesis System, Roche Applied Science). All the required equipments, reagents and procedures were provided and executed by Roche/NimbleGen.

### Design and production of the Coffea ssp. Nimblegen® custom array

Arrays were designed using sequence information available at the Brazilian Coffee Genome Project, which contains sequences of around 33 K genes identified in EST libraries prepared from different physiological and metabolic situations [31]. The *Coffea* dataset was composed by quality-filtered contigs from different non-normalized ESTs/cDNA libraries of two coffee species *Coffea arabica, Coffea canephora* and *Coffea racemosa,* and by singlets of this assembly. Only sequences with at least one blast hit against NR database (e-value <1e-10) were used as source sequences to generate probes for the 12 coffee microarray. The probes were designed by Roche-NimbleGen software, which selected unique sequences regions for each gene to avoid multiple hybridization with gene family members. Each microarrays consisted of 135.000 probes with length of 48 nucleotides and Tm average from 68°C to 76°C, representing 22,000 genes, with a minimum of 6 probes/gene. The final probe list was submitted to Roche-NimbleGen, Inc. (Madison, WI, USA) for quality control and subsequent probe array layout. Additional probes were also included on the microarray by Roche-NimbleGen, Inc. for quality control of the hybridization process. Microarray manufacture was synthesized *in situ* by photolithography on glass slides using a random positional pattern by NimbleGen (http://www.nimblegen.com/).

### Normalization and statistical analysis

Hybridized-microarray slides were imaged with a high resolution array scanner (GenePix 4000B Microarray Scanner, Molecular Devices Corp., Sunnyvale, CA, USA) and fluorescent signal intensities from each spot were quantified using NimbleScan Software (NimbleGen Systems Inc.). The intensity values were normalized using the oligo package from R statistics software [48]. The workflow used to normalize our data was followed as explained by the package provided for Nimblegen® expression microarrays. Fold change values were calculated comparing resistant and susceptible genotypes with and without infestation. All clusters were annotated using the blast2go software [48] in order to label them with their probable molecular function, biological process and cellular component. An automatic pipeline using Perl scripts was created to map each probe to its corresponding gene and annotation.

Differentially expressed genes (fold change values between 2 and -2) were identified using linear models and by taking into account technical and biological replicates. When individual probes met the criteria that average signals from resistant versus susceptible genotypes differed significantly by at least two fold, probes were selected for final analysis.

Functional characterization of differentially expressed genes was performed using Blast2GO [49] and also through directed searches on Gene Ontology (http://www.geneontology.org), KEGG (http://www.genome.jp/kegg) e InterPro (http://www.ebi.ac.uk/interpro/) databases.

### Microarray validation

Validation of selected differentially expressed genes was performed by real-time PCR. Gene-specific primers were designed using Primer Express 3.0 (Applied Biosystems) and Premier Primer 5.0 (Premier Biosoft International, Palo Alto, CA, USA). Gene sequences were aligned with GeneBank reference sequences using the tBLASTx tool. Possible ORFs and functional and conserved domains were identified using the *Open Reading Frame Finder (ORF FINDER)* and CDD tools from the NCBI database. In order to guarantee gene-specificity and avoid amplification of multigene families, primers were designed upon target regions which included the conserved domain and/or motif and anchoring outside the conserved region. A list of designed primers is shown on Table 6.

**Table 6:** Primer sequences used for validation in qRT-PCR analysis

**Name**	**Blast SOL network genomics**	**Annotation**	**Foward sequence**	**Reverse sequence**
0U17	NM	Protein	ACTACCAACATTCACAGCAGCTC	TTAACCCTGTTGAAGGTTAGTGC
0U6	SGN E1316291	Acid phosphatase	CTAATTAACCCTCTCCGCATGAT	GCCAACTCAGGCAATTATATACG
0U14	SGN E667484	Protein	TAGTCAAGAATATGGGCATGGAC	ATACCTTCTTGATTCACGCCTTC
0U2	SGN E1352064	Caffeine synthase	AAAGGGAGCATTTACTCTTCCAAAG	AGCATGCATCCTGAGAAATGTGGTA
0U4	SGN E1310344	Metallothionein-like protein	ATTCGTCTGCTCTGTGAAGATGT	ATACATGTTTCCGCAGTTTCCT
0U322	SGN E450221	nadp-dependent d-sorbitol-6-phosphate dehydrogenase	CCTTTGTGGCTTCTAAGCAAAT	GGAAAGCAGAGATTGACAAACAG
0U1	SGN E628893	No hits	CAAGGAAGATGCTTTTGACGAT	TGTAATTATGCTGCTGGTGCTAC
0U1	SGN E628893	No hits	CATTTAGTTTGGAAGGGGACAA	GGATACAGCCGGTAGGACTAACT
0U42	SGN E636199	---NA---	ACCCGCCGGGAAACC	GATGCACAGACAGGAATCACAAC
0D6	SGN E661762	---NA---	TTGGTAATGATGGAAGTGTCCTC	GCCAGTAATGGGATTGTAGTTGA
0D10	SGN E1033676	Glycerol-phosphate acyltransferase	CTAGCTTGACCAGGAAAGACAAG	GACTCAGGACTGCTCATTTCATT
0D2	SGN E676870	Protein	GGCAACTACTGCATTCTATCAGC	AAATGGATGAGCTGAAGGAGAAC
0D1	SGN E1320843	Protein	ACTAGTACTGGGTGTTGCCTCAA	GGTGAGCAAATAGTTGTTGTTGC
0D19	SGN E673783	Isocitrate lyase	GGCCAGGAGCAACAGACATT	ATTCTCTCACAATCTTGACTTTGCA
0D3	SGN E1320843	Zinc finger	CTGATTACGTCCGCTATCTCATT	AACCTATCGGACCTGTACCTGTT
0D4	SGN E1325444	Hypothetical protein	TACTGGCACTAATGGAGGAAATG	AATGGGGACAGATGTATCATCAC
0D8	NM	Glycerol-3-phosphate acyltransferase 6	TTCAAGAGTTTGGTACTGACGTG	ATCATGGTCTGTCTCTCGATCTC
0D9	SGN E1321887	gdsl-motif lipase hydrolase-like	TCTTAACTGGACTTCCTCCAATG	CCATTGAAGTTTAGAGCCACAAC
0D7	NM	Hypothetical protein	CCAAGACAGTTGATCTCCCTCTA	CGTAGTAGCTAGATGGTGCCAGT
0U9	SGN-E1337775	Organ-specific protein	GGTTTCTTTAGGGTTTCCTTCCT	CACAGTGTGTGTGTTTTGTTCCT
0U11	SGN-E1352070	Caffeine synthase	CCTAGCAAGCCATTTTGGAG	ATTCTTGGCAAACCTGTGGA
0U6	SGN-E1316291	Acid phosphatase	ATTACGGCTATGGCAGAATTAGC	CACCATGTTTCCTTGTTTGAGA
0U3	SGN-E1326397	Acidic endochitinase se2	CAGCAAATTCTTCCCTATGTCC	CAGCGTTTCAGGGTTAACATAAG
0U8	SGN-E1327615	Kunitz trypsin inhibitor	CTCTTCCTTTCATTTCTGCTCTTC	GACGTAGTACTCGACACCAGGAC

Corresponding cDNAs were synthesized from 250 ng of total RNA using the RevertAidTM Minus First Strand cDNA Synthesis Kit (Fermentas) according to the manufacturer protocol. PCR products were amplified using primers designed with Primer Express 3.0 (Applied Biosystems) and analyzed by Premier Primer 5.0 software (Premier Biosoft International, Palo Alto, CA, USA). In order to confirm primer specificity and presence of single amplicons, all PCR products were analyzed through a dissociation curve, with temperature varying from 60°C to 95°C.

Thermocycling and fluorescence detection were performed using ABI Prism 7300 Sequence Detection System (Perkin-Elmer Applied Biosystem). Real-time PCR amplification was carried out in a final volume of 15 μl by reaction using equal amounts of cDNAs (2 μl - 200 ng/μl) as template, 0,2 μM of each primer and 7,5 μl of Maxima SYBR Green/ROX qPCR master Mix (fermentas, USA) at the following conditions: 50°C for 2 min, 95°C for 10 min, 45 cycles of 95°C for 2 min, 62°C for 30 seg, 72°C for 30 seg. Data was collected during extension fase. Three independent qPCR reactions were performed for final quantification.

Expression levels of GAPDH were used as endogenous control. Relative gene expression was calculated using the 2^-ΔΔ*C*T^ method (where CT is threshold cycle) [50]. The Pearson correlation coefficient of linear regression from 18 pairs of microarray/qPCR expression ratios was calculated to validate the qPCR analysis.

## Competing interests

The authors declare that they do not have any non-financial competing interests political, personal, religious, ideological, academic, intellectual, commercial or any other competing interests.

## Authors’ contributions

JCM and DCC carried out experiments of infestation and the molecular genetic analysis by qRT-PCR. Both authors contributed equally for the experiments. PFG carried out the metabolic pathways analyses. ROV and MFC participated in the microarray analyses and performed the statistical analysis. MPM, LP, OGF performed the design and coordination of the study. JCM, DCC and MPM wrote the manuscript. All authors read and approved the final manuscript.
